# Education Research: Integrating AI-Enabled Interactive Case-Based Learning in a Preclinical Neurosciences Course

**DOI:** 10.1212/NE9.0000000000200351

**Published:** 2026-08-20

**Authors:** Tamara B. Kaplan, Kaiying Wang, Oliver Bichsel, Stephen Bacchi, Galina Gheihman

**Affiliations:** 1 Department of Neurology, Mass General Brigham, Boston, MA;; 2 Harvard Medical School, Boston, MA;; 3 Department of Medicine, Adelaide Medical School, Australia;; 4 Department of Neurology, Lyell McEwin Hospital, Elizabeth Vale, Australia; and; 5 Department of Neurosurgery, University Hospital Zurich, Switzerland.

## Abstract

**Background and Objectives:**

Case-based learning is central to neuroscience education, yet traditional approaches provide limited opportunities for iterative, interactive practice and individualized feedback among large preclinical cohorts. Large language models (LLMs) may support case-based learning by enabling interactive patient simulations and scaling formative feedback. Feasibility studies have shown high accuracy and minimal hallucinations; however, few have examined their real-world integration within medical school courses. We introduced AI-enabled cases as consolidation exercises in a preclinical neurosciences course. In this prospective pre-post observational study, we evaluated learner engagement, perceived educational value, and compared examination performance of students with access to AI-enabled cases compared with the prior year.

**Methods:**

Seven cases were developed and customized to the preclinical neurosciences course at Harvard Medical School. Cases were delivered through an online AI-enabled learning platform (TEACHABLE). All second-year students enrolled in the course completed weekly interactive cases in which they obtained history, examination findings, and investigations by questioning an LLM-simulated patient. Students then answered short-answer questions focused on clinical reasoning to reinforce course concepts. Responses were automatically scored and students received AI-generated feedback. Evaluation consisted of user engagement data, postcourse survey responses, and a comparison of midterm and final examination performance in AY26 (intervention year) vs the prior year (AY25).

**Results:**

A total of 172 students completed 1,236 cases and posed 23,310 questions to the LLM. Among 123 survey respondents (71.5%, 123/172), 77% agreed or strongly agreed that the cases consolidated their understanding of neurologic conditions, and 78% favored implementation of similar cases in other courses. Student feedback identified that they valued case interactivity and real-time feedback. Mean midterm scores were greater in AY26 (n = 172) at 85% (SD 7.3%) compared with 82% (SD 7.8%) in AY25 (n = 168) (*p* < 0.001). Mean final examination scores were similar between AY25 (88%, SD 5.7%, n = 168) and AY26 (89%, SD 6.7%, n = 172), with no statistically significant difference (*p* = 0.13).

**Discussion:**

LLM-supported interactive case-based learning offered a feasible, scalable, and well-received method for enhancing clinical correlation in preclinical neuroscience education. The approach maintained educational value and high-quality formative feedback without increasing faculty workload. These findings may be applicable in settings beyond preclinical neurosciences education.

## Introduction

Case-based learning (CBL) is a cornerstone of contemporary medical education, particularly in neuroscience, where students must integrate complex neuroanatomical, physiologic, and pathophysiologic concepts into coherent diagnostic reasoning.^1^ At Harvard Medical School (HMS) and many other institutions, CBL is implemented using structured case-based collaborative learning (CBCL), a pedagogy grounded in theories of constructivism, self-regulated learning, and experiential learning. CBCL is deliberately organized around 3 phases of learning: preparation before class, collaborative case discussion during class, and postclass consolidation exercises, which are essential for reinforcing key concepts, supporting reflection, and promoting long-term retention.^2^

Consolidation exercises represent a critical but challenging component of the CBCL framework. In many preclinical courses, consolidation takes the form of short-answer questions that ask students to articulate mechanisms, pathophysiology, or diagnostic reasoning. When faculty review and grade these responses, the process is time-intensive and difficult to sustain at scale.^3^ As a result, consolidation exercises are often ungraded and intended primarily for self-reflection, which limits the feedback students receive on their reasoning. This creates a gap between the pedagogical importance of consolidation and the practical constraints of delivering timely, individualized feedback in large preclinical cohorts.

These challenges are particularly salient in neuroscience education. Students frequently report difficulty connecting foundational concepts to real-world clinical presentations, and they often seek additional opportunities to practice applying knowledge in clinically meaningful contexts.^4^ Traditional consolidation exercises, while conceptually aligned with CBCL, may not fully meet learners' desire for authentic clinical integration or provide sufficient feedback to support deliberate practice.

Large language models (LLMs) such as OpenAI's GPT-5 and Anthropic's Claude Sonnet have emerged as compelling tools to support case-based learning.^5^ Their ability to generate natural language, simulate patient responses, and deliver tailored feedback raises the possibility of providing students with interactive deliberate practice beyond what faculty can routinely offer.^6^ The current educational literature demonstrates that LLMs can accurately simulate patient encounters, respond to diagnostic questions, and generate clinically plausible details when anchored to curated case scripts.^7^ Such approaches have been tested in neurology, internal medicine, and perioperative medicine contexts.^7-9^ For instance, prior neurology-focused studies showed that LLM-generated feedback on written reasoning could be benchmarked successfully against attending neurologist responses.^7^ Similarly, a feasibility study in perioperative medicine demonstrated that when cases were grounded in a structured screenplay, LLM question-answer pairs were both medically appropriate and devoid of hallucinations in more than 99% of interactions, although noting the relatively small sample size in such feasibility studies.^8^ LLMs may also have the potential to diversify cases, enhance realism, gamify learning, and provide rapid iterative cycles of question and feedback.^10,11^ However, few studies have examined the integration of LLM-supported learning activities into required medical school courses, nor have they evaluated their role specifically as consolidation exercises within an established CBL curriculum.

To address this gap, we used an online, freely accessible, AI-enabled learning platform called TEACHABLE (Transforming Education And Clinical Healthcare through Agent-Based Learning and Evaluation). The platform was developed by educators as a curricular innovation to fill this gap by providing structured clinical cases with integrated LLM-driven feedback on learners' written responses.^7^ TEACHABLE cases are informed by Knowles' Adult Learning Theory^12^ and Kolb's Experiential Learning Cycle.^13^ Cases are self-directed, relevant to medical practice, and may be completed individually or collaboratively. Within the platform, learners interact with simulated patients and then complete targeted short-answer questions linked to core learning objectives. LLM-generated feedback provides immediate, specific guidance on learners' reasoning, allowing for iterative refinement and deliberate practice^14^ without adding to faculty grading burden. Earlier studies prototyped TEACHABLE for neurology cases,^7^ but its use as a curricular-scale consolidation tool had not been evaluated.

The HMS Mind, Brain, and Behavior (MBB) course, a required rigorous preclinical neuroscience block, offered an ideal educational setting in which to evaluate LLM-supported case-based learning in a preclinical environment. In MBB, AI-supported cases were introduced specifically as consolidation exercises at the end of the week, following the week's in-class CBCL sessions. In this implementation, the TEACHABLE framework allowed students to interact with AI-simulated patients through history, examination, and investigation queries, with the goal of supporting clinical correlation. However, rather than entering an assessment and plan, a structure used in prior implementations, students completed multiple short-answer questions (SAQs) at the end of each case that matched learning objectives at the preclinical course level. These SAQs targeted diagnostic reasoning and key discriminating features, and were automatically scored by the LLM, which provided tailored written feedback.

### Hypotheses

We hypothesized that implementing LLM-supported interactive cases as consolidation exercises would be feasible, highly engaging for students, perceived as educationally valuable, and possible without increasing faculty workload. Accordingly, this observational study aimed to evaluate the real-world implementation of AI-supported, SAQ-based consolidation exercises within a required preclinical neuroscience course, focusing on learner engagement, perceptions of educational value and acceptability, and feasibility from a faculty workload perspective.

## Methods

### Study Design, Setting, and Participants

This prospective pre-post observational implementation study was conducted during the 2025–2026 academic year at Harvard Medical School. Second-year medical students enrolled in the required Mind, Brain, and Behavior course participated in weekly interactive cases delivered through the TEACHABLE LLM platform. All 172 students in the course had access to the cases, and participation, though strongly encouraged, was not mandatory. Students were also allowed to repeat cases if desired. We assessed feasibility and student satisfaction and acceptability with this student cohort. We also compared midterm and final examination scores in the AY26 (academic year 2025–2026) students (intervention cohort) compared with the scores of the prior AY25 (academic year 2024–2025) students who served as the control year. Examination structure and blueprint domains were consistent across academic years, with minor routine refinements to individual items.

### Case Development

Case development followed the TEACHABLE screenplay methodology, previously assessed for accuracy in pilot studies in neurology and perioperative contexts.^7,9^ Seven cases were intentionally selected to represent high-yield neurologic syndromes aligned with weekly course objectives. Each case included a clinical vignette mapped to course content, structured history and examination components, and targeted investigations designed to reinforce core neuroscience concepts.

The 7 cases used in the MBB course were developed by the TEACHABLE study team and the course director, modified from prior case frameworks, and independently peer reviewed by a second neurologist for clinical accuracy, alignment with learning objectives, and appropriate level of difficulty for preclinical learners. Effective deployment required thoughtful case design, faculty review, and periodic quality assurance review of AI-generated feedback by the course director.

Table summarizes the presenting neurologic syndromes, diagnostic reasoning targets, and core neuroscience concepts reinforced by each case.

**Table:** Educational Scope and Reasoning Focus of AI-Enabled Consolidation Cases

Presenting neurologic syndrome	Case title	Primary diagnostic reasoning focus	Core neuroscience concepts reinforced	Final diagnosis
Visual disturbance	Right eye vision loss	Localization to optic nerve and differentiation of inflammatory and vascular causes of acute visual loss	Optic nerve and visual pathway anatomy, inflammatory demyelination	Optic neuritis
Episodic loss of consciousness	Funny turn	Distinguishing seizure from syncope and transient ischemic events using history and semiology	Seizure pathophysiology and antiseizure medication mechanisms of action	Focal epilepsy with impaired awareness (post-stroke epilepsy)
Sudden-onset focal neurologic deficits	Right-sided weakness	Neuroanatomic localization and identification of stroke subtype based on clinical features	Corticospinal pathway, relevant neuroanatomical structures, vascular territories	Lacunar stroke
Acute severe headache	Severe headache	Recognition of red flags and differentiation of primary vs secondary headache syndromes	Meningeal inflammation, intracranial pressure, infectious mechanisms	Bacterial meningitis (Neisseria meningitidis)
Gait disturbance	Shuffling gait	Differentiation of neurodegenerative vs medication-induced movement disorders	Basal ganglia circuitry, dopaminergic pathways, motor control	Drug-induced parkinsonism
Progressive behavioral and cognitive change	Behavioral change	Syndromic localization and differentiation of frontal vs subcortical cognitive disorders	Frontal lobe networks, executive function, neurodegeneration	Frontotemporal dementia (behavioral variant)
Generalized weakness	Weakness	Distinguishing upper vs lower motor neuron processes and identifying neurodegenerative patterns	Motor neuron anatomy, neuromuscular physiology, neurodegeneration	Amyotrophic lateral sclerosis

Cases were written in a text format and uploaded to the TEACHABLE platform. The TEACHABLE platform^15^ uses an LLM to simulate interactive patient encounters. Students were required to obtain information by asking questions across the domains of history, examination, and investigations. The LLM responded to these queries by drawing from the established case screenplay, supplemented by medically appropriate generative additions when relevant, an approach that prior feasibility testing had shown to be effective and accurate when models are properly constrained.^8^ Students could enter either text-based or voice-based questions. The LLM patient simulator could respond through text or audio. The cases at this stage did not have any video, soundtrack, or imaging elements integrated.

After completing their interactive information gathering, students answered 3 to 4 SAQs. These questions focused on clinical reasoning: identifying the most likely diagnosis, specifying key supporting clinical features, explaining underlying pathophysiologic mechanisms, articulating red-flag features, and selecting appropriate next investigations. These SAQ focused the learner on clinical correlation of preclinical concepts learned in the class that week, reinforcing core topics. The SAQ-based format was selected to align with assessment practices used within the MBB course.

Each SAQ was automatically scored by the LLM using a structured rubric developed by neurology faculty. Feedback was provided instantaneously, highlighting areas of strength, identifying missing or incorrect reasoning, and recommending specific aspects of the case or underlying basic science content for review. Students were allowed to revise and resubmit their SAQ responses, enabling an iterative cycle of feedback and improvement.

### Quality Assurance Review of AI-Generated Feedback

During implementation, the course director conducted periodic quality assurance review of AI-generated feedback. This review was intended to monitor for major inconsistencies, clinically inaccurate feedback, misleading guidance, or unsafe recommendations. The review was performed by the course director rather than by multiple independent faculty raters and did not involve structured line-by-line review of all student responses and AI-generated feedback across the 1,236 completed cases. Feedback quality was therefore monitored as part of implementation quality assurance but was not formally rated using a standardized rubric in this study.

### Intervention Evaluation

#### Student Engagement

Automatic platform analytics recorded the number of case completions, total questions asked to the AI, time spent per case, and SAQ submission patterns.

#### Student Perceptions of Value

A 31-item end of course survey is administered after each course in the HMS preclinical setting. Two items were added to this survey to evaluate student perceptions of the cases, focusing on the educational value and potential for expansion into other areas of the curriculum. Free-text comments also solicited open responses from students about their perceived value of the platform. Students were invited to complete the surveys as part of their routine course evaluation.

#### Comparison of Examination Performance Before and After Implementation of AI-Enabled CBL

Student performance on the midterm and final examinations was compared between 2 academic year cohorts: AY25, which occurred before the implementation of AI-enabled case-based learning, and AY26, which occurred after its introduction. Examination scores were summarized using means, SD, and medians for each cohort. Differences in mean scores between cohorts were assessed using the Welch independent-samples *t* test. Effect sizes were estimated using Cohen *d*. All statistical tests were two-sided, with statistical significance defined as *p* < 0.05.

### AI Disclosure

GPT5 was used to aid in the writing of this manuscript to aid with clarity. Gemini was used to aid in the production of figures. The authors have read and take responsibility for the content of the manuscript.

### Ethical Approval

The project was reviewed and approved by the HMS Program in Medical Education's Educational Scholarship Review Committee. The Committee determined that the project was designed to inform educational quality improvement, did not meet the definition of human subject research, and therefore did not require formal Institutional Review Board review.

### Data Availability

Data collected in this study are available from the authors on reasonable request.

## Results

### Student Demographics

Demographic data were not collected from participants. Participants were second-year medical students enrolled in a required preclinical course at a United States medical school.

### Student Engagement

Students engaged extensively with the interactive case platform. Over the duration of the course, students completed a total of 1,236 cases or approximately 7.2 cases per student. This indicates that some students repeated cases. Overall, students posed 23,310 questions to the LLM. This represented an average of approximately 19 questions posed per student per case. The questions posed encompassed targeted history collection, focused neurologic examination inquiries, and investigation requests.

Every student who completed a case also submitted responses to the short-answer questions. Typically, each case involved 3–4 SAQs, and many students completed iterative revisions after receiving formative feedback. In free-text responses, students reported using the feedback to refine their diagnostic reasoning process, identify gaps in understanding, and reinforce conceptual links across neuroscience topics.

### Student Perceptions of Value

**Figure 1:**
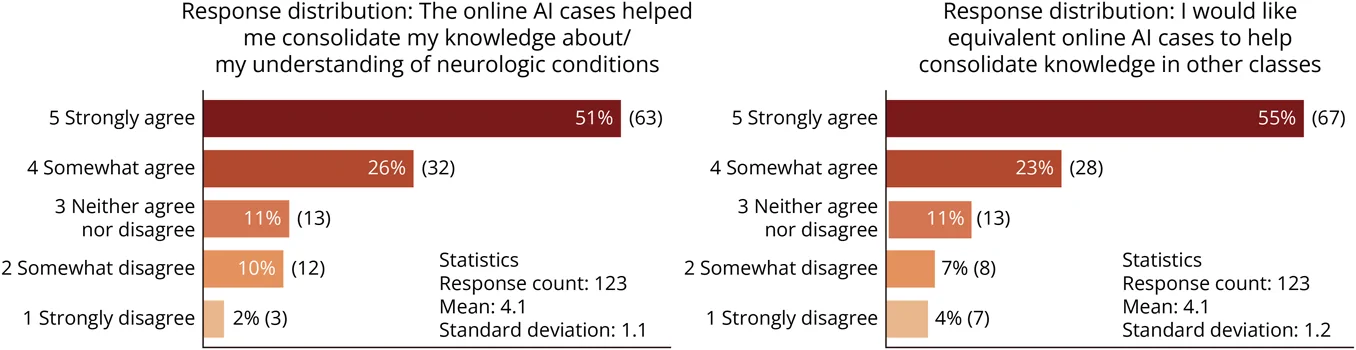
Postcourse Evaluation Survey

A total of 123 students of 172 (71.5%) completed the postcourse evaluation survey. Survey responses demonstrated strong learner support for the educational value of the AI-supported cases (Figure 1). Among 123 respondents, 51% strongly agreed and 26% somewhat agreed that the cases helped consolidate their understanding of neurologic conditions. Only 2% strongly disagreed. When asked whether they would like equivalent AI-supported cases implemented in other classes, 55% strongly agreed and 23% agreed, with only small minority expressing disagreement.

**Figure 2:**
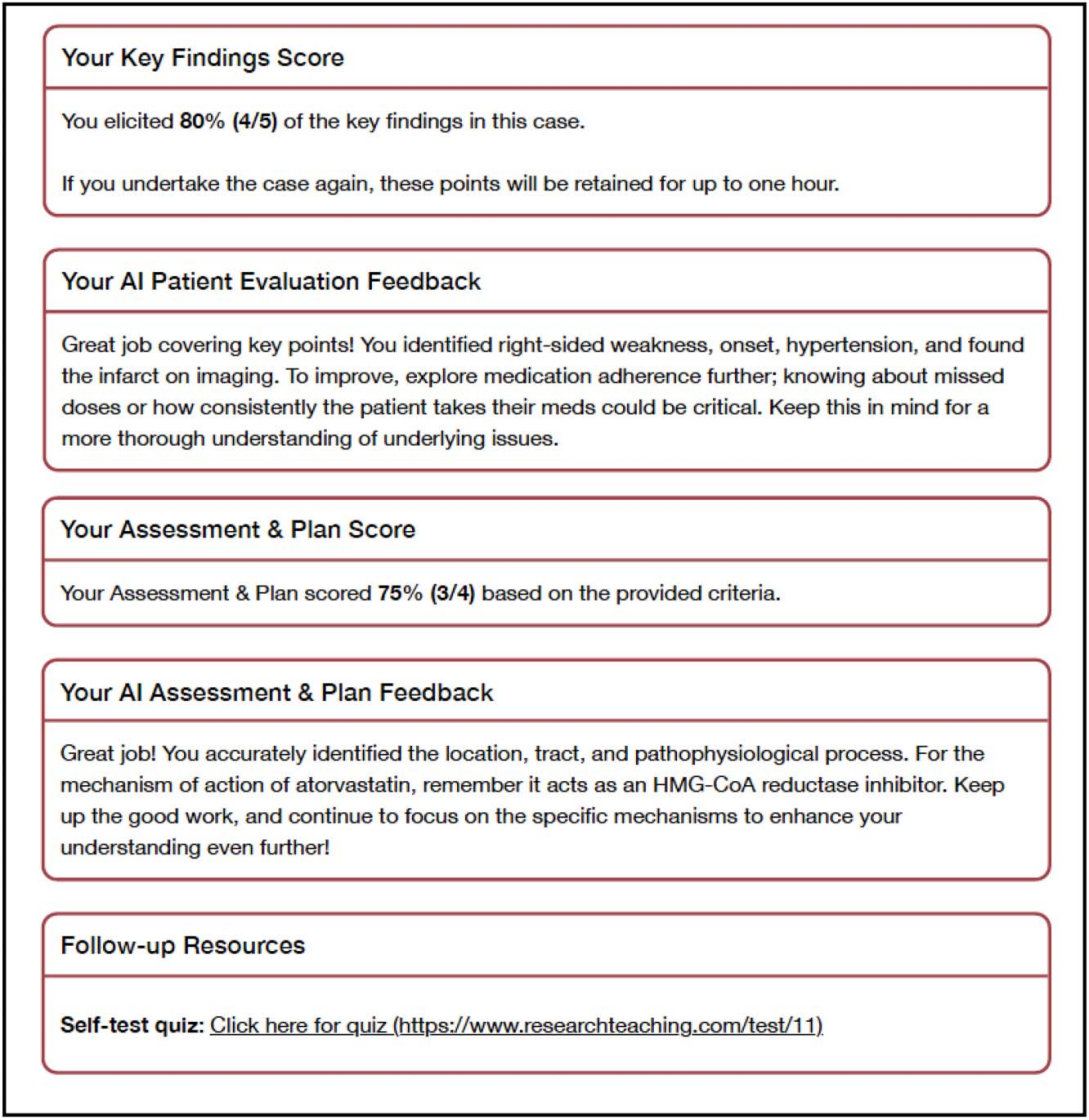
Example of AI-Generated Feedback Provided After Completion of an Interactive Case The platform provided the learner with scores and individualized feedback based on the key clinical findings elicited and the submitted assessment and plan, followed by links to additional learning resources.

Free-text comments from students highlighted the usefulness of immediate targeted feedback, the engaging nature of interactive patient questioning, and appreciation for the opportunity to practice diagnostic reasoning in a low-stakes environment. Students frequently noted that the cases helped bridge the gap between basic neuroscience content and real clinical presentations. They valued the ability to query the AI patient to clarify pertinent positives and negatives, an approach that felt authentic to clinical encounters. Many commented that the iterative feedback on SAQs offered a level of granularity and personalization not previously available in large preclinical courses. An illustrative excerpt of the feedback is provided in Figure 2.

### Examination Performance

Mean midterm scores were greater in AY26 (n = 172) at 85% (SD 7.3%) compared with 82% (SD 7.8%) in AY25 (n = 168) (*p* < 0.001). This difference was statistically significant using the Welch independent-samples *t* test (mean difference 3 percentage points, *p* < 0.001, Cohen d ≈ 0.4). Median scores were 86% in AY26 compared with 84% in AY25, supporting a shift in overall performance. Mean final examination scores were similar between AY25 (88%, SD 5.7%, n = 168) and AY26 (89%, SD 6.7%, n = 172), with no statistically significant difference (Welch *t* test, *p* = 0.13, Cohen d ≈ 0.16). Median scores were identical in both cohorts (89%).

### Implementation Experience

The implementation did not increase faculty grading demands. After adoption of LLM scoring, faculty time shifted from grading of student responses to upfront case development and periodic quality assurance review by the course director. Initial faculty effort was required to create cases aligned with the weekly learning objectives. During implementation, the course director periodically reviewed samples of AI-generated feedback, and no major inconsistencies or unsafe feedback were identified during these quality assurance reviews. However, as described in the Methods, feedback quality was not independently rated by faculty using a structured rubric in this implementation study.

## Discussion

In this prospective pre-post observational study, the integration of LLM-supported interactive cases as consolidation exercises within a required preclinical neuroscience course effectively augmented case-based neuroscience education with an opportunity for iterative, deliberate practice and individualized feedback delivered at scale. Students engaged deeply with the cases, generated large volumes of targeted clinical questions, and rated the educational value of the experience highly, expressing interest in similar consolidation activities across other courses. Importantly, replacing traditional faculty-graded or ungraded consolidation exercises with LLM-scored patient interactions and SAQs preserved educational rigor while substantially reducing faculty workload.^11^

The results reinforce several emerging themes in the literature regarding the growing role of LLMs in supporting case-based learning, particularly when used to strengthen the consolidation phase of instruction. Prior work has shown that LLMs can accurately simulate patient interactions and generate clinically coherent responses when grounded in curated case scripts, as seen in pilot studies in both neurology and perioperative contexts.^8,9^ Our study extends this work by demonstrating that LLM-supported cases can be integrated feasibly and effectively into a required preclinical course specifically as consolidation exercises, rather than as stand-alone simulations or experimental learning activities.

Adapting TEACHABLE for this purpose required reconfiguring the assessment structure from a full assessment-and-plan format to SAQs that align more closely with preclinical learning objectives and goals of consolidation practices. While this required some upfront investment by the course director to ensure alignment between the cases, learning objectives, and assessments, it enabled authentic consolidation of in-class content through clinically situated reasoning tasks with immediate feedback. A key lesson learned is that broader applicability will require course leaders to make some modest adjustments to existing cases, enabling customization to learner level, local curricular goals, and clinical realities such as differences in available diagnostic tests or local epidemiology.

Second, the use of LLMs to generate individualized written feedback can support diagnostic reasoning by highlighting strengths, identifying misconceptions, and encouraging reflective practice. As noted in commentary by Stretton and colleagues, this feedback-driven interactivity can enhance learner engagement and provide diverse case exposures that would be difficult to generate manually.^10^ In the context of consolidation exercises, LLM-generated feedback offers a scalable mechanism to deliver consistent, structured guidance aligned with case-specific learning objectives, addressing a long-standing challenge in large preclinical courses where faculty feedback may be variable, delayed, or absent. Unlike human assessment, LLMs can consistently apply the same evaluative framework without being affected by fatigue, cognitive bias, or variability in interpretive standards, which may enhance the reliability of formative feedback at scale.^11,16^ Immediate, individualized feedback also supports self-directed learning by enabling rapid cycles of revision and self-correction, approximating conditions of deliberate practice that are difficult to achieve consistently in clinical environments.^17^Our findings build on prior work demonstrating the usefulness of LLM-generated feedback in nonclinical settings, including multiple-choice assessments and writing instruction,^18,19^ and align with emerging evidence that learners perceive AI feedback as accessible and understandable while continuing to value human feedback for judgment, context, and trust.^20^Incorporating LLM feedback alongside expert oversight in a hybrid format could optimize learning efficiency, standardize evaluations, and foster equitable access to high-quality formative assessment.^8,11^ No major inconsistencies or unsafe feedback were reported during these audits, and the course director perceived the feedback to be accurate, consistent, and aligned with course objectives. This observation is consistent with findings from earlier TEACHABLE neurology prototypes, which indicated that LLM feedback could closely match attending neurologist explanations.^21^

This study extends prior work by demonstrating that LLM-supported CBL can be embedded successfully into a required course with high learner satisfaction. The SAQ-based format provided a standardized and curriculum-aligned assessment structure that reinforced neuroanatomic localization and pathophysiologic reasoning. Engagement metrics indicated substantial interaction rather than superficial participation, with students completing more than 1,200 cases and posing more than 23,000 questions. The average of approximately 20 questions per case suggests hypothesis-driven inquiry, a core component of diagnostic reasoning. Iterative refinement of SAQ responses after receiving AI-generated feedback mirrors deliberate practice models and supports self-directed learning.

The observed improvement in midterm performance in AY26, coupled with similar final examination outcomes across cohorts, is noteworthy, although in the context of a prestudy/poststudy design. Students exposed to AI-supported consolidation performed better earlier in the course, while end-of-course achievement remained comparable. This pattern suggests that the curricular modification may have influenced the timing of learning rather than the ultimate level of mastery. One possible interpretation is that structured, feedback-driven consolidation facilitated earlier acquisition or integration of foundational neuroscience concepts. Final examination performance was similar across cohorts, suggesting no observable shift in overall summative achievement or reduced assessment rigor and instead supports the possibility of earlier consolidation. However, because this was not a randomized comparison and cohorts may differ in unmeasured ways, these findings should be interpreted cautiously. Furthermore, there may be a “ceiling effect” with baseline high performance on final examinations among this cohort, making it challenging to see an impact on learning from our intervention. In future studies, we hope to assess other markers of clinical reasoning beyond the final examination to determine whether engagement with TEACHABLE cases can predict an increase in traces of clinical reasoning among students.

Medical students' positive engagement with AI-supported consolidation aligns with prior literature demonstrating generally favorable learner attitudes toward AI in medical education.^22^Surveys and systematic reviews across multiple settings show that students perceive AI-enabled learning tools as efficient, personalized, and supportive of real-time feedback, particularly when integrated thoughtfully into structured curricula.^23-25^ Our findings contribute to this literature by demonstrating feasibility within a required neuroscience course and by suggesting a potential shift in learning trajectory rather than final performance outcomes.

Despite these promising findings, limitations remain. This is a prospective pre-post observational implementation study without a parallel control group, limiting causal inference regarding examination performance because of the potential for confounding. Feedback quality, though perceived as helpful, was not independently rated by faculty in a structured manner in this implementation study. Periodic quality assurance review was conducted by the course director, but this did not involve systematic line-by-line review of all student responses and AI-generated feedback across the 1,236 completed cases. AI-generated scoring and feedback were not formally compared with expert human feedback using a structured rubric, nor were inter-rater agreement or systematic error analyses performed as part of this implementation study. As a result, conclusions about the accuracy, consistency, safety, and educational quality of AI-generated feedback should be interpreted cautiously. Our recently published companion study addressed this question more directly and provides a dedicated evaluation of LLM-generated narrative feedback compared with expert faculty feedback.^26^

Not all students completed the postevaluation survey. There may have been response bias among students who favored the platform compared with those who did not. Cases were developed to match curriculum content and priorities for the MBB course and may not be directly applicable or generalizable to other medical schools or non-neurosciences courses. However, we demonstrate that with upfront faculty investment, cases can be adapted to and customized for local needs. Model updates over time may introduce variability. As with all LLM applications, careful oversight, content auditing, and clear communication with students about limitations are essential to avoid overreliance or uncritical acceptance of AI-generated content.^6,8,11^

Future studies could investigate objective performance of students in the clinical setting, evaluate differential impacts across student subgroups, and explore applications in clerkship-level neurology education. Such studies should include objective measures of learning and should be conducted across diverse sites to improve generalizability. In addition, future studies should formally evaluate the quality, accuracy, consistency, safety, and educational utility of AI-generated feedback through comparison with expert faculty feedback, assessment of inter-rater agreement, and systematic analysis of feedback errors or omissions. These analyses should include structured review for inaccuracies, omissions, misleading feedback, and unsafe recommendations. Longitudinal evaluation of the nature of questions asked by learners would also be informative because this may provide insight into how learners' clinical reasoning progresses over time and how feedback is incorporated. We hope other sites will consider implementing TEACHABLE cases and evaluating their impact on student learning across diverse institutions, providing external validation, and strengthening the credibility of our findings. The integration of multimodal LLMs capable of interpreting imaging may further enhance case realism and expand the skill sets addressed. We also hope to expand the opportunity for AI-enabled case-based learning to other preclinical classes to determine generalizability outside the neurosciences setting.

This study demonstrates that LLM-supported interactive case-based learning can be successfully implemented at scale as consolidation exercises within a preclinical neuroscience course. Students engaged robustly with AI-simulated patient interactions, valued the immediate AI-generated feedback structure for SAQs, and expressed strong support for expanding such cases into additional courses. The approach introduced a novel, scalable formative feedback model that delivered educational value without expanding faculty grading workload. These findings support the role of LLMs as effective partners in delivering individualized case-based learning and structured formative feedback in preclinical neurosciences education.

## Glossary

CBCL: case-based collaborative learning
CBL: case-based learning
HMS: Harvard Medical School
LLM: large language model
MBB: Mind, Brain, and Behavior
SAQ: short-answer question
